# Expression of CD56 isoforms in primary and relapsed adult granulosa cell tumors of the ovary

**DOI:** 10.1186/1746-1596-3-29

**Published:** 2008-07-09

**Authors:** Hans-Ullrich Völker, Sabine Engert, Andreas Cramer, Melanie Schmidt, Ulrike Kämmerer, Hans-Konrad Müller-Hermelink, Stefan Gattenlöhner

**Affiliations:** 1 University of Würzburg, Institute of Pathology, Würzburg, Germany; 2 University of Würzburg, Dept. of Gynaecology, Würzburg, Germany; 3 Missionsärztliche Klinik, Dept. of Gynaecology, Würzburg, Germany

## Abstract

**Background:**

Adult granulosa cell tumors of the ovary (GCTs) are sex cord stromal tumors of unpredictable behaviour. Up to now, the prediction of the relapsing/malignant potential remains difficult. CD56 (NCAM) in GCTs was previously described in only two studies. However, the expression of its isoforms was not examined.

**Methods:**

30 GCTs (16 primaries, 14 relapses) were investigated immunohistochemically with antibodies against Pan-CD56 (CD56^Pan^) and the isoform with 140/180 kDa length (CD56^140/180 kDa^). The reaction was assessed with respect to percentage of positive cells and intensity of staining.

**Results:**

In all GCTs, CD56^Pan ^was expressed, but differences were found between primaries and relapses. The percentage of CD56^Pan ^positive tumor cells was lower in relapses, whereas CD56^140/180 kDa ^showed a higher staining intensity in the latter.

**Conclusion:**

Expression of CD56 is an additional sensitive and helpful immunohistochemical tool for histopathologists diagnosing a GCT. It does not seem possible to provide a validly individual risk assessement. However, the different expression of CD56 isoforms might indicate important changes in the course to a more malignant behaviour.

## Background

Adult granulosa cell tumors (GCTs) of the ovary common are sex cord stromal tumors of unpredictable clinical behavior. They account for 2–5% of all ovarian neoplasms [1]. The reported 5-year survival is 75–90% in FIGO stage I, 55–75 % in stage II, and 22–50 % in stages III/IV [2,3]. An important problem for therapeutic decisions apart from surgery is the unpredictable course of disease. Considerable efforts have been undertaken to predict the risk of relapse or metastasizing. Correlations between more malignant behavior and patients' age, menstrual status, incomplete surgery, mitotic count, or proliferative activity have been reported [3-6]. The influence of mutated cell cycle regulatory proteins like p53 or other molecular changes remained unclear [7-11]. So far, reliable parameters are not defined.

CD56 is expressed in adult neural, neuroectodermal, and neuroendocrine tissue and different tumors [12] as neuroendocrine tumors, plasmocytomas, or melanomas. Its expression identifies a subgroup of tumors with an unfavorable prognosis e.g. in myeloid leukemia, adenoid cystic carcinoma, squamous cell carcinoma, or renal cell carcinoma [13-16].

CD56 is a membrane-bound cell surface sialoglycoprotein and a member of the immunoglobulin supergene family which induce cell-to-cell interactions during embryonic development, cell migration, and organogenesis [17-19]. Three main isoforms with molecular weights of 120, 140, and 180 kDa are known. These are generated from a single gene by alternative splicing. At least 20 major exons contribute for encoding these different isoforms, and further small exons can give rise additional isoforms [17]. The appearance of the 140/180 kDa isoform was found to be associated with a higher degree of malignancy [20].

In GCTs, only two studies are available which report an expression of CD56 [21,22]. An analysis of its isoforms was not performed up to now.

## Methods

### Specimen

30 primary and relapsed GCTs of 19 patients (surgery between 1996 and 2007) were investigated. 16 GCTs were primary tumors, 14 were relapses. Eleven relapses of 4 patients were available for direct comparison with the primary. Three further relapsed GCTs were initially diagnosed and treated loco alieno, unfortunately the specimen of their primaries were not disposable for this study. Medical records of all patients were available. The follow up time ranged from 24 months to 16 years.

### Staining

After surgical resection, the specimen were formalin fixed and paraffin embedded. 2 μm sections of the routinely processed paraffin blocks were stained with hematoxylin-eosin (HE) for histopathological diagnosis. Only cases with typical morphology were included. Proving the diagnosis, all cases were stained with vimentin (Mouse, V9, 1:400, DAKO) and inhibin (Mouse, R1, 1:40, Serotec) and found positive.

Immunohistochemical stainings for CD56 were performed in the usual immunoperoxidase technique (Kit: Advance HRP, DAKO). Following antibodies against were used: CD56^Pan^, which recognizes all isoforms (Mouse, 1BC, 1:40, Novocastra), and CD56^140/180 kDa ^(Mouse, NCAM-OB11, 1:500, Sigma). Additionally, Ki67 (MIB-1, 1:200, Dako) was stained. Only areas with antigen integrity (vimentin+, inhibin+) were evaluated. The minimal size of representative areas was 1.5 × 1.5 cm. One sample per cm tumor diameter was investigated, the median values were gathered for calculation.

### Analysis and Statistics

Immunohistochemical reactions with CD56 were discriminated in a weak (1), moderate (2), or strong (3) staining intensity. The intensity was assessed by comparison with a strong reaction in the positive controls (small cell neuroendocrine carcinomas of the lung). The percentage of stained tumor cells was determined semiquantitatively.

All data were analyzed using Microsoft Office Excel^® ^and SPSS^®^. The descriptive statistical values, i.e. average, median, minimum, maximum, and standard deviation/standard error were computed. Furthermore, the significance of differences was tested by Chi-Square test resp. Mann-Whitney-U-test.

## Results

### Clinical data

Table 1 shows data of patients. 11 patients were premenopausal, 8 postmenopausal at time of first surgery. 12/19 (63.2%) primaries were free of relapses over 36 months up to 16 years. Relapses occurred 30 months up to 8 years after initial diagnosis, no more than four relapses/patient were found.

**Table 1:** Clinical data at the time of first surgery

	**Primaries with relapse**	**Primaries without relapse** **during observation period**
**Age [years] ** **(Average, Min, Max)**	55.1 (23–78)	57.3 (34–85)
**Menopausal at first** **surgery**	4 premenopausal 3 postmenopausal	7 premenopausal 5 postmenopausal
**Tumor size [cm] ** **(Average, Min, Max)**	13.4 (2.5–26)	7.8 (4–25)
**FIGO stage**	5* FIGO I 2* FIGO II	8* FIGO I 4* FIGO II

In the cases (primaries or relapses) investigated here, the primary tumor stage was in 11 cases FIGO I (IA:7, IB:1, IC:1) and in 6 cases FIGO II (IIA:4, IIB:2). In 2 cases, the FIGO stage was not exactly documented, but the clinical data correspond with FIGO I.

Between primary and relapsed GCTs, patients' age, first tumor stage (mostly FIGO I), or size of primary did not differ significantly.

### Conventional parameters

The proliferation (Ki67) was 4.5% (1–10) in primaries and 11.3% (2–40) in relapses (P < 0.05). Moreover, the relapses harbored a significant higher number of cases with a proliferation above 10% (3/16 vs. 6/12; P < 0.0001). However, the proliferation was not different between primaries with and without relapse (4.0%, 1–5 vs. 4.6%, 1–10). The mitotic index per high power field (HPF) did not differ significantly between primaries and relapses (2.0/HPF, 0–10 vs. 3.3/HPF, 0–17) resp. between the two groups of primaries (1.75/HPF, 1–3 vs. 2.0/HPF, 0–10).

### Expression of CD56

**Figure 1:**
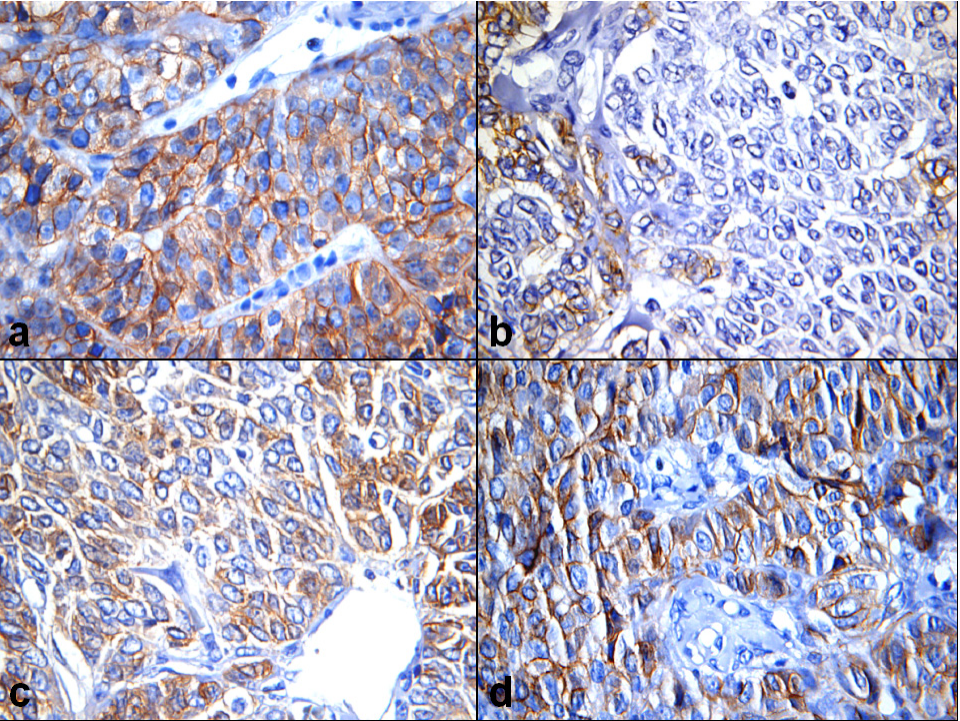
Examples for CD56 staining (Immunoperoxidase ×400). A) Strong expression of CD56^Pan ^in nearly all tumor cells in a unrelapsed case, B) Weak reaction with the same antibody in only few tumor cells of a relapse (4^th ^relapse of another case), C) Expression of CD56^140/180 kDa ^in a relapse, and D) Strong expression of the same in nearly all tumor cells of its primary.

**Figure 2:**
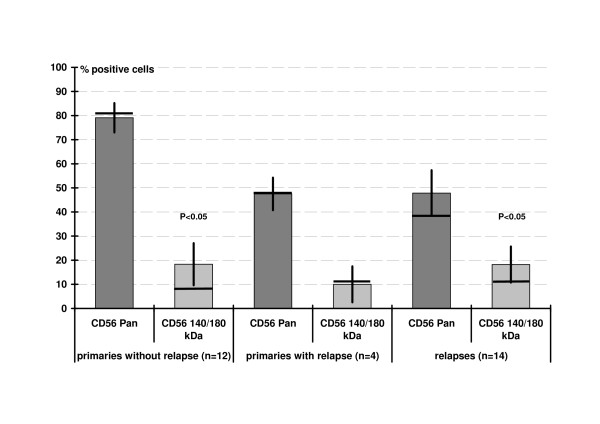
Percentage of positive stained tumor cells (average with standard error, bar: median).

**Figure 3:**
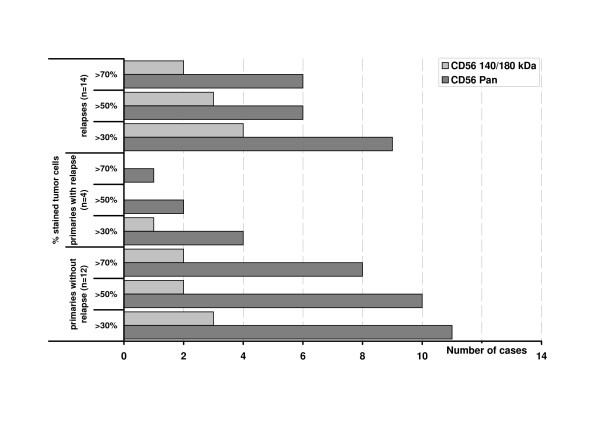
Number of cases distributed in three groups (>70%, >50%, >30% positive cells). No significant differences between primaries with and without relapse and relapses.

All tumors, both primaries and relapses, stained positive for CD56^Pan^. 9/16 primaries (56.3%) reacted with CD56^140/180 kDa^, 8/14 relapses (57.1%) were positive. In primaries, the median of positive cells for CD56^Pan ^was 80% (5–100) and 5% (5–80) for CD56^140/180 kDa ^(P < 0.05). Relapses showed a median of 35% (5–100) positive cells in CD56^Pan ^and 5% (0–80) for CD56^140/180 kDa ^(P < 0.05). Figure 1 gives examples for staining, figure 2 indicates these data in comparison of primaries without relapse, primaries with relapse and the relapsed tumors. The percentage of positive tumor cell classified in three groups with >70%, >50%, and >30% positive cells showed no significant differences (figure 3).

**Figure 4:**
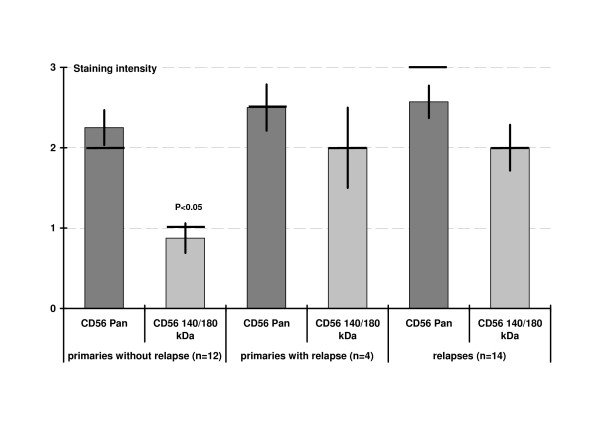
Staining intensity (1-weak, 2-moderate, 3-strong) in the different groups of granulosa cell tumors (average with standard error, bar: median). No significant differences, but trend to a higher staining intensity in primaries with relapse and relapses. Significant lower staining of CD56^140/180 kDa ^in primaries without relapse (see also figure 2).

Positive cells in unrelapsed primaries showed a significant lower staining intensity for CD56^140/180 kDa ^compared with CD56^Pan^. In relapses and their primaries, the staining intensity was higher than in unrelapsed primaries (figure 4).

CD56^Pan ^was strongly expressed in 7/16 primaries and 10/14 relapses, CD56^140/180 kDa ^in 1/16 primaries and 4/14 relapses. The differences were not significant.

## Discussion

The expression of CD56 (neural cell adhesion molecule, NCAM) in adult granulosa cell tumor of the ovary (GCTs) was previously described in two studies [21,22]. The expression of CD56^Pan ^and the isoform CD56^140/180 kDa ^was not investigated up to now.

We investigated 16 primaries and 14 relapses (of together 7 primaries). The number is not high enough for comprehensive statistical evaluation, but seems adequately for insights in expression profiles for CD56 in these rare tumors.

GCTs express CD56 constantly. This finding constitutes a further diagnostic tool for the surgical pathologist apart from inhibin or vimentin. In difficult cases, the differential diagnosis of GCT may be provided by positivity of CD56 in discrimination e.g. of poorly differentiated carcinoma or endometrial stromal sarcoma [21]. However, expression of CD56 alone can not prove a GCT because of the possibility of positive reaction in a lot of other malignant tumors [12-16].

Human granulosa cells of pre-ovulatory follicles and thecal cells have been detected to express CD56 [17,22]. Similar to inhibin or activin, CD56 is a regulator of growth and differentiation in ovarian folliculogenesis [23]. Thus, it is understandable that GCTs express CD56. It seems to be an important factor involved in the recognition and intercellular interaction of ovarian endocrine cells and participates in the regulation of the cyclic remodeling processes of the ovarian endocrine compartments [18].

The major function of CD56 is the homophilic binding NCAM-NCAM [16]. In clusters of granulosa cells of follicles, CD56 was found by Mayerhofer et al., whereas cells devoiding CD56 spread out and form monolayers [18]. CD56 is thought to favor the development of metastases by supporting cell dissociation processes [16,24,25]. We found a less number of tumor cells positive for CD56^Pan ^in primaries with relapse and relapsed GCTs (not significant, but clear trend). Loss of CD56 could be interpreted as a sign of dedifferentiation during the tumor progression and with respect to the binding function of CD56 to a loosening of cell adhesion. Differences in the percentage of positive tumor cells in GCTs were also reported by Ohishi et al., but not analyzed in this matter [21].

In several malignant tumors, CD56 expression predicts a more aggressive biological behavior [13,14,16,24,26,27], especially in presence of the 140/180 kDa isoform [20]. In GCTs, we found a more frequent appearance of strong expression of CD56^140/180 kDa ^in relapsing primaries and relapses in comparison to unrelapsed primaries. This shift to the high molecular isoform could be interpreted as a hint for the more aggressive biological behavior of relapsing cases. However, the findings of expression in this small cohort seem sufficient for refusing a predictive value of CD56 expression, because of the heterogenous distribution.

Increased mitotic count and proliferation were associated with relapsing as reported before [2,28,29]. However, these markers are also not suitable for prediction of behaviour, because we found only differences between primaries and relapses, but not between relapsing and unrelapsed primaries. Another conventional factors reported associated with a good prognosis are low FIGO stage, small tumor size (<10–15 cm), and unruptured tumor during surgery [2]. However, even though FIGO stage is thought as an important criterion, the tumor stage does not give valid information regarding the prognosis, since the majority of GCTs is diagnosed at stage I [2].

## Conclusion

CD56 is constantly expressed in adult granulosa cell tumors of ovary. Its expression is most probably determined by tumor histogenesis. Therefore, apart from other immunohistochemical markers like inhibin, the detection of CD56 in GCTs is a helpful diagnostic tool for the histopathologist in difficult cases. However, an individual prediction of clinical behavior via expression of CD56 isoforms is not possible.

## Competing interests

The authors declare that they have no competing interests.

## Authors' contributions

HUV, HKMH, SG Preparation of cases, immunohistochemical investigation, analysis of data, SE, AC, MS analysis of cases, all clinical parts, discussion, UK statistical analysis.
